# Behavior of Isolated Disturbances Superimposed on Laminar Flow in a Rectangular Pipe^1^

**DOI:** 10.6028/jres.064A.027

**Published:** 1960-08-01

**Authors:** Grover C. Sherlin

## Abstract

An investigation was conducted in a horizontal transparent rectangular pipe to study the behavior, in laminar flow, of an isolated turbulent-like disturbance produced by injecting a quantity of dye into the pipe 39 feet from the entrance. As the resulting mass of colored water moved downstream, time-distance measurements were made for the front of the dye mass and for the rear of the disturbance. The experimental setup, which is described in some detail, permitted reasonable control over the mean flow rate from which Reynolds number was calculated. The utilization of the data unfolded a functional relationship among three quantities: The ratio of the velocity of the rear of the disturbance to the velocity of the front of the dye *U_R_/U_F_*; the distance from the origin, *X_F_*; and the Reynolds number *R.* The similarity of this work to that being done by Lindgren in Stockholm is mentioned.

## 1. Introduction

The investigation described herein is the outcome of what was originally intended to be a study of turbulent motions in pipe flow using dye techniques in water. While it turned out to be difficult to learn much about the turbulence itself by this method, it was possible to study the initiation of turbulence and to distinguish between turbulent and nonturbulent points of the stream. The investigation was therefore turned to a study of these phenomena, specifically to the observation and study of a disturbance which when introduced at a given point propagated downstream and either died out or developed into an isolated slug of turbulent flow.

The fact that flow in a pipe can be intermittently laminar or turbulent has been known for many years. Only recently, however, with the work of Rotta [1]^2^ and Lindgren [2,3] has the intermittency been portrayed in the light of downstream-convected regions of turbulent fluid separated by regions of laminar fluid. The turbulence in such cases originates sporadically at various locations in the pipe, and the regions are distributed randomly and occur with increasing frequency as the Reynolds number becomes greater. With a sufficient distance downstream from the origin of a disturbance and at a Reynolds number above some critical value the turbulent regions have grown together and the flow has become continuously turbulent. Similar phenomena are presumed to occur in smooth conduits of any cross-sectional shape.

The purpose of the investigation reported here was to study the development and propagation of a single turbulent region which had a known origin in time and space and could thereafter be followed and observed over a long course. Interest in the behavior of such a region arises primarily from the fact that it is presumably a fundamental link in the process by which flow becomes turbulent.

The observations were made in a long rectangular pipe with a well-faired entrance. An effort was made to reduce ambient disturbances so as to raise the limiting Reynolds numbers for laminar flow to as high a value as possible. The observations were made only in the region where there appeared to be fully developed pipe flow. The set of consecutive observations on a single disturbance will be called a “run”.

## 2. Apparatus

### 2.1. Rectangular Pipe and Constant-Level Supply Tank

**Figure 1:**
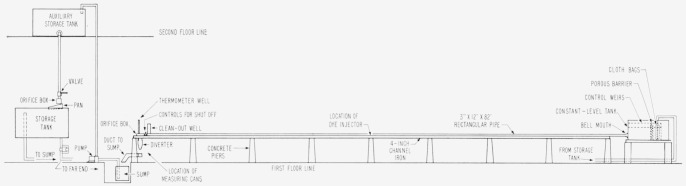
Layout of major components of the circulating system.

A circulating system using water from the city mains was constructed in the summer and fall of 1953. The system consisted of a constant-level supply tank, a horizontal rectangular transparent pipe, discharge controls, and tanks for storage and for regulation of flow. A simplified layout of the major components is shown in figure 1.

The constant-level tank was a rectangular-sided steel vessel 36 in. by 48 in. by 75 in. in size. A rectangular hole near the bottom led into the horizontal pipe through a plastic bellmouthed connection 33 in. long.

Water was delivered to the tank through two short lengths of vertically standing perforated pipes. A cloth bag about 10 in. in diameter and 36 in. long was fitted over each pipe to provide a fairly uniform distribution of flow. To improve the flow distribution, a porous barrier about 4 in. thick, made of ⅝ in. glass spheres, was formed across the tank 42 in. upstream from the bell mouth. Above the water level and near the bellmouth was mounted a hook gage graduated in thousandths of a foot.

The horizontal transparent pipe was 82 ft long. Internally it was 3 in. high and 12 in. wide. It was constructed of acrylic plastic sections approximately 68 in. long. Irregularities at the junctures of the sections were kept to a minimum. The pipe was supported 45 in. above the laboratory floor by two parallel rails of 4-in. channel iron which were suspended across concrete piers placed in a straight line at 9-ft intervals.

At 3-ft intervals along the length of the plastic pipe, 12-in. lengths of stainless steel tubing, 0.008 in. inside diameter, were inserted through the top and bottom walls on the centerline. Nigrosine black dye flowed by gravity from a reservoir into the tubing. Through a hole in the wall of each piece of tubing the dye issued into the water, moved along with the flow, and produced a straight horizontal black line when the flow was laminar. Simple packing glands on the pipe wall prevented leaks and permitted adjustment of the dye streamers in the vertical plane.

At a point 39 ft from the entrance of the pipe a special dye injecting device was located for the purpose of creating a disturbance to the water flow. The essential features of this device were a toy balloon that could be filled with a measured volume of red dye, a 6-in. length of 1½-in. plastic tube to confine the balloon when under pressure, and a cylindrical lead weight to force the dye out of the balloon. Rubber tubing connected the balloon with a ⅛-in. pipe nipple set flush in the bottom of the rectangular plastic pipe on the centerline.

From the location of the injection device to the end of the plastic pipe, vertical lines were drawn on both sides of the pipe at 1-ft intervals. These lines were used to mark the passage of dye masses along the plastic pipe.

### 2.2. Discharge Instrumentation

At the discharge end of the pipe were controls for shut-off, a thermometer well, an orifice box, a diverter, measuring cans, and ducts to carry water to a sump for recirculation.

The controls for shut-off were a manifold arrangement of five 1¼-in. brass gate valves that were connected at each opening, through short nipples, to corresponding holes in two 3 by 12 by ⅛-in. brass plates. One plate was screwed to the end of the rectangular pipe, the other plate to the upstream side of the orifice box.

Glued to the top of the orifice box was a vertical transparent plastic pipe that served as a thermometer well for a 59° to 115° F thermometer, graduated in twentieths of a degree. The bottom of the box was a brass plate with 15 symmetrically placed orifices, in five sizes of 116-, ⅛-, ¼-, ½-, and 1-in. diam, respectively. The three orifices of each size were spaced equidistantly on one of five concentric circles. Below each orifice was brazed a short nipple of sufficient inside diameter to allow free exit of water. Each nipple was covered with a pipe cap when not in use.

The divert er was directly below the orifice box. It was used to obtain samples of water for weighing to determine the mean velocity in the pipe. The water-carrying components of the diverter were constructed of acrylic plastic; the structural supports, bearings, and other parts were made of metal. A ball bearing mounted on the moving arms of the diverter actuated momentarily a microswitch as the arms shifted through the central or neutral position in directing the water flow to either side of the transparent pipe.

The water to be weighed was collected in galvanized cans, 15 in. long, 6 in. wide, and 14 in. deep, as it flowed from the diverter. Two plywood cars, one on each side of the transparent pipe, each held 15 cans as well as a special container which was placed under the diverter to receive water at the beginning or end of an experiment. The special containers each had a discharge spout at the bottom leading through duct work into the sump. During the experimental run, sample cans on alternate sides of the transparent pipe were successively moved into position under the diverter spout until all were filled. Each time the diverter was shifted to spill water into a different can, the microswitch on the diverter was actuated and a pulse was transmitted to the time-interval pen in a single-channel recorder. Thus, as the measuring cans were filled progressively with about 35 lb of water, the time required to fill each can was indicated by the distance between marks on the recorder chart.

### 2.3. Modification to Apparatus

After the apparatus had been set up, several modifications in the equipment and its use were found necessary in order to obtain the desired measurements. For example, to secure a smooth, steady flow of water through the system, an auxiliary storage tank was added on the floor above the main setup. Water flowed from this tank through an orifice box to a circular tank below, which was 96 in. in diameter and 54 in. high, and thence to the constant-level tank that supplied the transparent pipe. The orifice box permitted a reproducible regulation of the flow rate. Water issued from the box downward in jets which were converted to tranquil horizontal flow after the jets impinged on a large horizontal flat pan in contact with the water surface in the circular tank. An overflow pipe in the circular tank helped maintain the water level so that while observations were being made a steady flow of water under constant pressure was delivered to the constant-level tank. A 2-in. galvanized pipe, controlled by a parallel arrangement of a 2-in. and a ¾-in. gate valve, connected the circular tank to the constant-level tank.

In the constant-level tank the flow of water over the control weirs at first generated secondary currents which distorted the flow pattern through the bellmouth. However, it was found that the quantity of water overflowing the weirs could be reduced to a mere trickle by carefully adjusting the supply valve. This trickle of water did not cause distortion of the flow pattern, but it did assure the observer that the water level in the tank and the pressure in the rectangular pipe were being kept very nearly constant.

Experience showed that when fresh water was added to the system to make up for overflow losses, a laminar flow regime would not develop so long as the water temperature varied by as much as 0.2° in 10 min. It was therefore necessary to circulate the fresh water through the system until a temperature equilibrium was obtained.

It was found that the black dye streamers were affected by the elevation of the flask that supplied the dye. If the flask was below a particular point, water from the rectangular pipe would enter the dye tube, whereas if the flask was too high, the dye would emerge as a jet and cause disturbances in the dye stream. To obtain sharp, straight streamers in regions of laminar flow, special supports were devised to permit careful adjustment of the elevation of the flask.

Throughout the study efforts were made to reduce or eliminate vibrations and noises that might disturb the water and set up regions of instability. However, within the range of flow rates at which laminar flow was obtained, normal vibrations and noises in the laboratory seemed to have less effect on the stability of laminar flow than did distorted velocity distribution at the pipe entrance, foreign matter or particles in the pipe, excessive temperature gradients, or possibly other unidentified factors.

## 3. Experimental Procedure and Observations

Five observers were used in the experiments. Three observers followed the progress of the slug of red dye and the resulting disturbance as the dye moved downstream. Each of the three marked the time of an observed event by a signal impressed on a single-channel recorder, the signal from each being attenuated a characteristic amount by series resistors for purposes of identification. A fourth observer recorded the temperature at approximate 30-sec intervals throughout the run.

As soon as straight, parallel dye streams throughout the length of the rectangular pipe indicated the presence of laminar flow, the sampling of water was begun by the fifth observer. Immediately following the filling of the second sample can, a measured quantity of red dye was squirted forcefully into the rectangular pipe by the dye injector. Then foot by foot the first observer marked, on the recorder, the passage of the red dye downstream. After 3 or 4 ft of travel, the front of the dye stream took on a parabolic or bullet-nosed appearance that was sharply defined in the vertical plane.

It was noticed that some time after the front end of the red dye passed each of the tubes supplying black dye, a fresh streamer of black dye would wave violently like a flag in a wind storm. The second observer recorded the time and location of this phenomenon for each fresh dye streamer as an indication of the passage of the front of the mass of disturbed water.

The third observer followed the rear of the disturbance, i.e., the boundary between sharp, straight dye lines upstream and the disordered dye lines downstream. He marked the time on the recorder for each foot this boundary progressed downstream. There was some uncertainty as to when the boundary first existed because the mechanism of turbulent regeneration in the particular rectangular pipe used in this investigation was a succession of pulses, each of which acted on the straight dye streamer to start a bulge at a point 6 or 8 in. upstream from the last irregularity in the dye streamer. The bulge would increase in amplitude and suddenly the whole region between the initial pulse and the previously disturbed mass downstream would appear to be identically disturbed. Although some of the straight dye streamers behind the disturbance were being distorted continuously, the rate at which this occurred was such that the whole disturbed mass of water moved downstream and could be followed to within a foot of the exit where the flow began to accelerate into the 1¼-in. openings. Eventually the disturbed mass of water passed on out the exit, leaving the flow in the pipe free from disturbance.

The collected water samples were weighed on a Howe beam scale. The density and viscosity of the water at the measured temperature were assumed to conform, within the limits of experimental error, to the values given in standard tables; consequently they were not measured.

It was originally planned to make triplicate runs for each flow rate studied. The three runs of each set were to differ from one another only as a result of using different control orifices that had the same nominal diameter but different locations in the orifice box. However, flow conditions in the transparent pipe were found to vary with the location of the control orifices in the box. It was therefore not feasible to make use of the triplicates in analyzing the data. Instead, some runs were repeated to obtain a measure of reproducibility.

## 4. Data Processing

### 4.1. Reynolds Number

It is well known that the parameter which characterizes the relative importance of viscous action in a moving fluid is the Reynolds number. It will be used in this paper to aid in physically classifying the 27 different runs and will be denoted by the symbol *R* as defined by

$$R=\frac{\rho d\bar{U}}{\mu },$$

where *ρ* = density, d=4 × hydraulic radius, $\bar{U}$=mean velocity in the pipe, and *μ* = absolute viscosity. The hydraulic radius is the ratio of the cross-section area, *A*, of the pipe to the wetted perimeter, *P*, so that d=4*A/P*.

The value of $\bar{U}$ was determined from weighed samples of water taken at successive time intervals. A fourth-degree polynomial was fitted to the data with the aid of SEAC to obtain *W=f*(*T*), where *W* is the total mass of water collected at the end of time *T*. The mean velocity was then obtained by

$$\bar{U}=\frac{dW/dT}{A\rho }$$

and the Reynolds number by

$$R=\frac{4\left(dW/dT\right)}{\mu P}.$$

The Reynolds number associated with the runs illustrated in this paper are mean values representing the conditions existing during the run.

### 4.2. Time-Distance Relations

**Figure 2:**
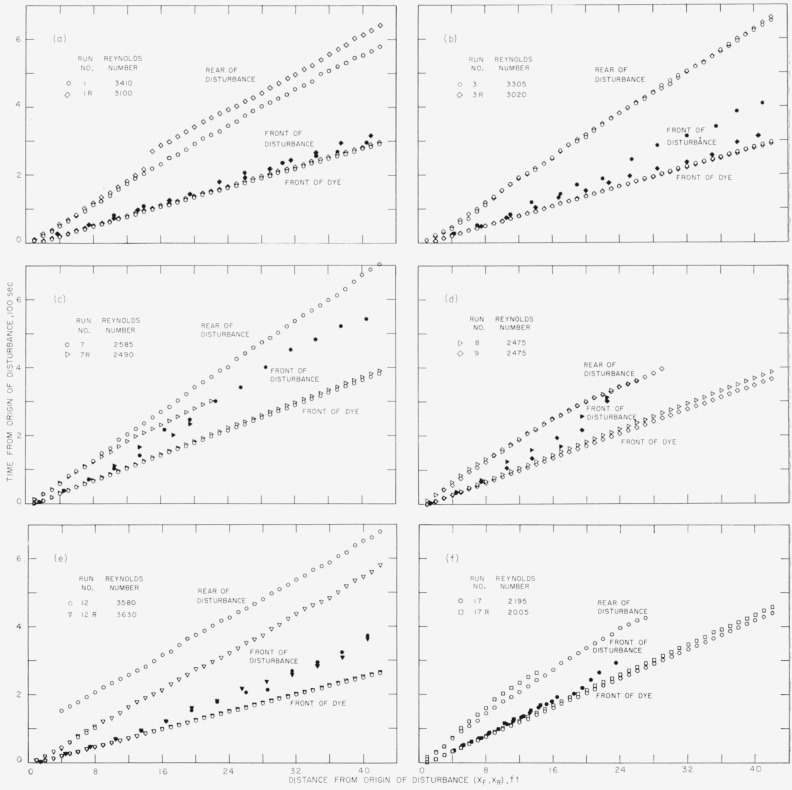
Time-distance relationships for twelve runs showing variations in behavior of an isolated disturbance superimposed on laminar flow.

**Figure 3:**
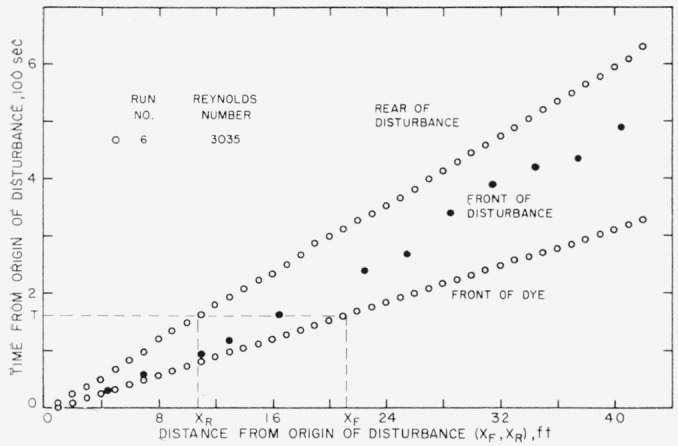
Time-distance relationship for a single run to show that at time *T* the region between *X_F_* and *X_R_* occupies the length (*X_F_* − *X_R_*).

To make use of the data obtained in these 27 runs, several relationships were considered for study. The simplest relationship, time and distance, was graphed for each of the runs with time as a function of distance traveled by the vertex of the parabolic-shaped front of the dye. Also time was graphed as a function of the distance traveled by the front of the disturbance, and by the rear of the disturbance. Some of these graphs are reproduced here in figures 2 and 3 to illustrate some charactersitics of isolated disturbances superimposed on laminar flow.

The several samples of the time-distance relationship in figure 2 illustrate the general behavior and the irregularities that at times appear. Common to all of the graphs shown for the front of the dye is the essentially straight line of points indicating that the velocity of the front of the dye was constant throughout each run. In contrast to this regularity, the graphs of the front and the rear of the disturbance varied considerably. For example, during run 1R a spot of spontaneous turbulence was noticed approaching the rear of the disturbance from upstream. The discontinuity in the graph marks the joining of this spot with the main turbulent slug, and the step in the curve is the time required for the spot to pass the observation point and leave the upstream part of the pipe free of disturbance. A larger step is seen to occur in run 12, this time from a spontaneous disturbance originating farther upstream from the injector-caused disturbance and accordingly producing a longer disturbed region and requiring more time to pass by the observation point.

Another feature is illustrated by an early termination of the data for the rear of the disturbance in certain runs. This signifies that disordered motions could no longer be observed and apparently had died out as in runs 7R, 8, 9, 17, and 17R. The phenomenon was generally associated with the lower Reynolds numbers, with run 7 appearing to be just enough above a borderline to escape the effect. The curves of figure 2 show that the behavior of the disturbances can be divided roughly into two types : Those at the higher Reynolds numbers where the rear of the disturbance moved at approximately a constant rate, and those at the lower Reynolds numbers where the rear moved at a rate changing with distance along the pipe.

### 4.3. Growth of the Disturbance

The primary purpose of obtaining the data illustrated in figure 2 was to determine what happened to the disturbance after it left the point of origin and progressed downstream through the pipe. By observing the dye streamers it was apparent that the disturbance produced a turbulent region consisting initially of the turbulent motions caused by the fluid injection. While the subsequent behavior was not completely consistent, as shown by figure 2, it was apparent that turbulent motions would persist causing the extent of the region to grow. Since growth in length was an indication of whether the motions were dying out or reinforcing themselves, the time-distance data were used to determine the length of the region as a function of the distance of travel and the Reynolds number.

By referring to figure 3, which is an example of a, run free from extraneous effects, one may see that at any given time *T* after the introduction of the disturbance the front of the dye is at a distance *X_F_* and the rear of the turbulent region is at a distance *X_R_* from the origin. Therefore at time *T* the region between the front and rear occupies the length (*X_F_−X_R_*).

The data for each run were smoothed by least-square computations on SEAC. The values of *T* as expressed by a fourth-degree polynomial in *X_F_* are denoted *T_F_*; *T_R_* is the similarly adjusted value of *T* as a function of *X_R_.* The adjusted values of *T* were used as a parameter to get a smoothed relationship between simultaneous vailles of *X_F_* and *X_R_*. Having obtained values of *X_R_* corresponding to values of *X_F_*, successive values of (*X_F_−X_R_*) could be determined for each of the integral values of *X_F_.* The successive differences of the values of (*X_F_−X_R_*) provided a measure of the change in length of the region for successive distances along the pipe, *X_F_.*

**Figure 4:**
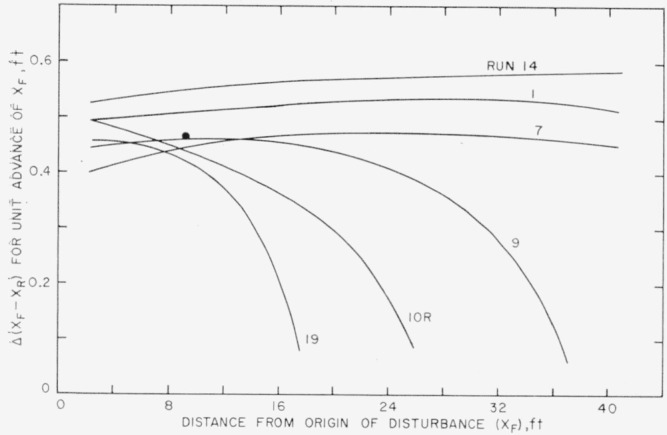
Selected runs to show that the relationship of the change in length of (*X_F_−X_R_*) to the distance traveled by the front, *X_F_*, suggests a system of curves.

Figure 4 is a summary plot showing how the change in length of the disturbed region Δ(*X_F_−X_R_*) varies with *X_F_* along the pipe. For run 14 the value of Δ(*X_F_−X_R_*) appears to approach some constant value as *X_F_* increases. There is uncertainty about the possible behavior of runs 1 and 7 beyond the range shown, but in runs 9, 10R, and 19 the quantity Δ(*X_F_−X_R_*) appears to approach zero as *X_F_* increases. This method of processing the data demonstrates experimentally that at the low rates of flow a disturbance will cease to grow, but if the flow rate is high enough, the disturbance will increase in length at a steady rate.

### 4.4. Velocity of Propagation of Disturbed Regions

**Figure 5:**
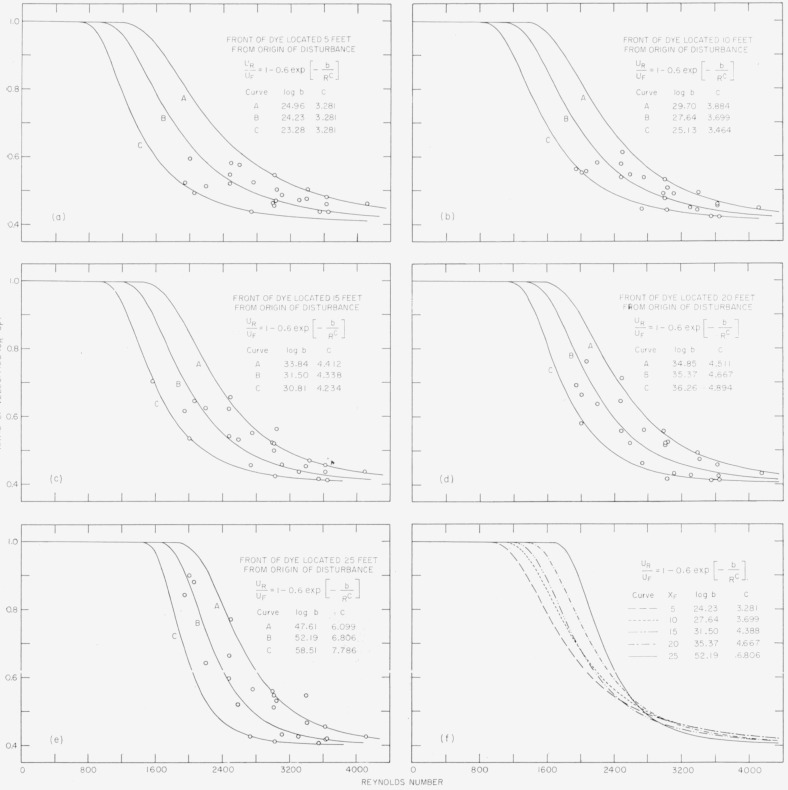
Sets of curves at 5-ft intervals of *X_F_* showing the development of a trend that suggests the curve fitting to eq (1); and showing the emergence of critical values when the “B” curves are brought together.

At this stage of the analysis, a mathematical functional relationship has not been found between *X_F_* and *T_F_*, between *X_R_* and *T_R_*, or between *X_R_* and *X_F_*; however, the relation has been expressed through tabulated data and by approximating polynomials. The derivatives of the polynomials, *dX_R_/dT_R_* and *dX_F_/dT_F_*, could be expected to give reasonably close approximations of the velocities *U_R_* and *U_F_.* The ratio of these velocities as a function of *R* was first considered for the location *X_F_*=5. When these were plotted, the wide scatter of points for the 27 runs formed no recognizable pattern. To explore the possibility that some recognizable order might be found to exist among these quantities as the disturbances moved downstream, computations were made for four additional locations along the pipe. These progressive relationships formed the patterns shown in figures 5(a) to 5(e).

While the scatter of the points was large, due mainly to the irregularities illustrated in figure 2, it was possible to draw curves that enclosed the majority of the points in each plot. These curves were extended beyond the Reynolds-number range of the experiment on the basis of the indicated trend, as *X_F_* increased, and on the assumption that at low Reynolds numbers the ratio of velocities would be essentially unity and at the higher Reynolds numbers the ratio would approach some constant value, such as 0.4, asymptotically. For each of the five locations along the pipe the initial envelope curves were sketched and then a curve of the following form was fitted to them:

$$U_{R}/U_{F}=1-0.6\;\exp\left(-b/R^{c}\right),$$ (1)

where the coefficient 0.6 was chosen to make curves of *U_R_*/*U_F_* approach the asymtotic value indicated in figure 5 and *b* and *c* were chosen after successive approximations. A representative mean curve (curve B in each plot) was computed using parameters that placed it approximately half way between the two envelopes. Where these “B” curves are collected in figure 5(f), a crossing over is seen to occur in the neighborhood of Reynolds number of 2,700, suggesting that this might indicate critical values. To explore this possibility, the particular values *R*=2,700 and *U_R_/U_F_*=0.51, were substituted in eq (1) to produce an equation containing *b* and *c.* By solving the equation for *b* and substituting in eq (1) again, the following relationship is obtained

$$\frac{U_{R}}{U_{F}}=1-0.6\;\exp\;\left[-\frac{{\left(2,700\right)}^{c}\;\log\;\left(0.6/0.49\right)}{R^{c}}\right].$$ (2)

**Figure 6:**
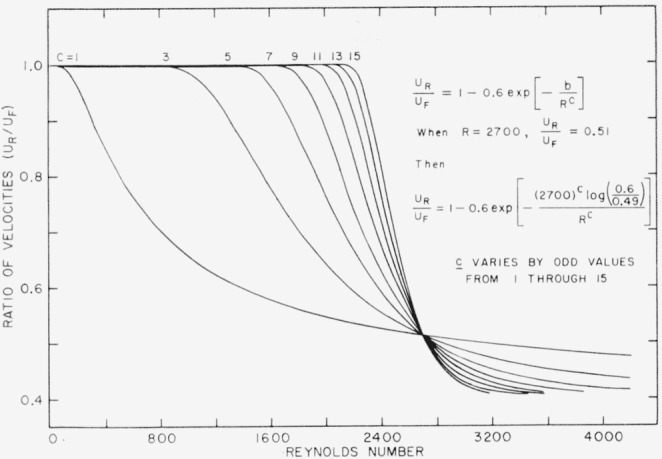
System, of curves resulting from eq (2) when the parameter “c” is varied through odd integral values.

Equation (2) has been plotted in figure 6 for odd integral values of *c* from 1 to 15. It turns out that the curves for *c*=3 and *c* = 7 approximate very closely the curves for *X_F_*=5 and *X_F_*=25 of figure 5(f), which suggests a functional relation of *c* and *X_F_.* Equation (2) may be rewritten without affecting the relationships to give

$$\frac{U_{R}}{U_{F}}=1-0.6\;\exp\left[-\log\;\left(0.6/0.49\right)\;\exp\;(c\;\log\;\left(\frac{2,700}{R}\right)\right].$$ (3)

**Figure 7:**
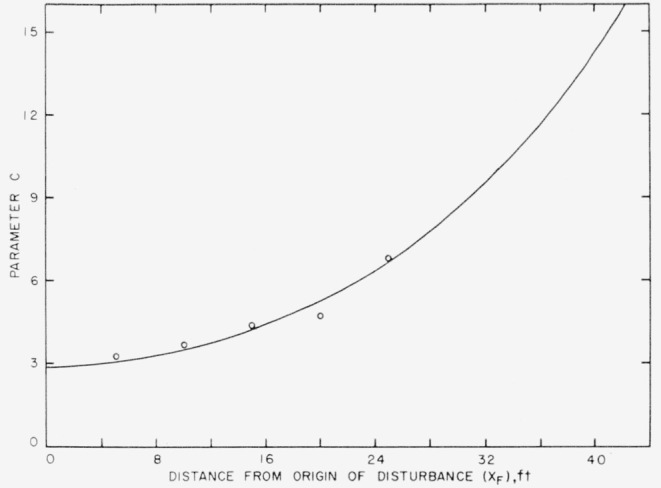
An extrapolation of the values of parameter “c” to cover the 42-ft range of *X_F_* in this investigation.

**Figure 8:**
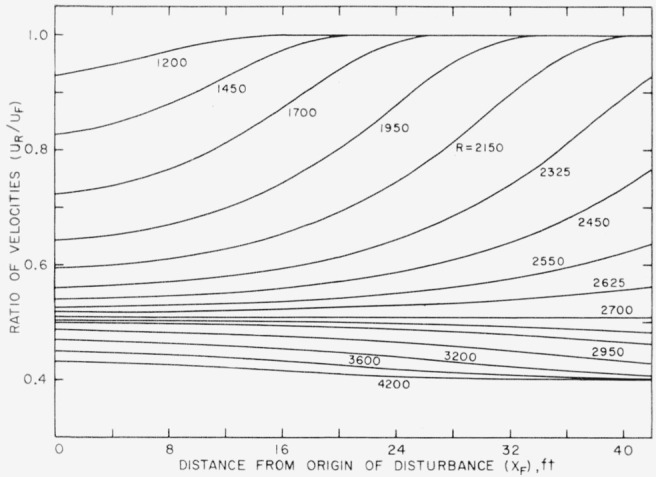
A system of curves computed for particular value of Reynolds number when for eq (3) the values of parameters “c” are taken from figure 7 at 2-ft intervals of *X_F_*.

This form of the equation was found to be useful in computing *U_R_/U_F_* when *R* is held constant and *c* varies as a function of *X_F_.* The tabulated values of figure 5(f) give a numerical relation of *c* to *X_F_* for five values of *X_F_.* These are plotted in figure 7 and a curve has been drawn as a reasonable interpretation of the behavior of the function in the extrapolated range. Using values of *c* taken from the curve for *X_F_* at 2-ft intervals, a system or family of curves was computed for 16 values of *R* from 1,200 to 4,200. These are the curves shown in figure 8.

Several features are at once evident from this family of curves. The lower the Reynolds number the more rapidly *U_R_*/*U_F_* approaches unity with distance downstream. For the Reynolds number 2,700 the straight line *U_R_*/*U_F_*=0.51 divides the field into two parts wherein the curves in the upper part approach unity and those in the lower part decrease to a lower value than that existing at the origin. These trends are taken to be an indication of changes in the vigor of the motions. For example, an upward trend of the curves, which indicates a faster downstream convection of the rear, is taken to mean a damping out of the disturbance.

**Figure 9:**
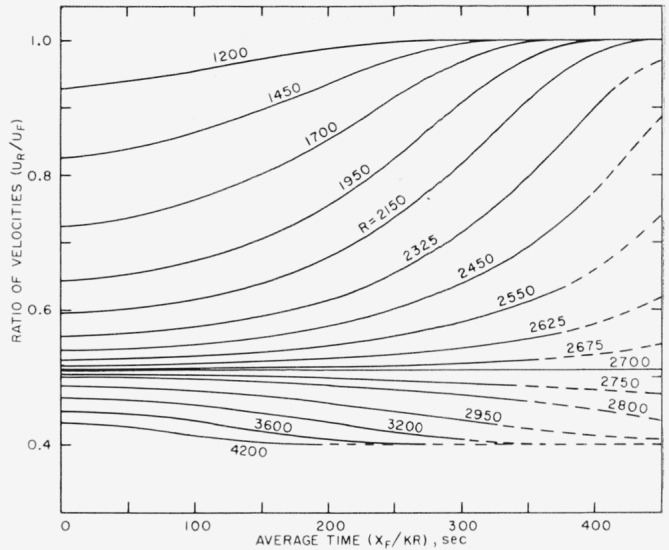
A system of curves differing from those of figure 8 by having for the abscissa an averaged time (*X_F_*/K*R*) where for each curve the values of K and *R* are constant.

To assist in the discussion to follow, these same phenomena have heen represented on an approximate time basis in figure 9. Mere *T_F_* is the averaged time taken by the front to travel to the point *X_F_.* The approximation, which for the intended purpose of the figure is assumed to be permissible, involved using a constant factor, K, to reduce Reynolds number to velocity of the front in each case.

## 5. Discussion of Results

Figures 8 and 9 permit certain inferences to be drawn about the behavior of turbulent disturbances in a pipe. First of all there is the not unexpected emergence of a critical Reynolds number above which turbulent motions once started will persist indefinitely and below which they will eventually die out. The value of 2,700 found here falls within the range of the values published in [4] for straight pipes of various cross section. More significant, however, from the standpoint of novelty is the behavior of a disturbance below and above the critical value. When a stirring motion (here termed the disturbance) is introduced at a very low Reynolds number, it quickly dies out. As the Reynolds number is increased, it persists longer and longer. This shows that there is a reinforcement of the motions which increases with Reynolds number and which, while not sufficient to balance the damping action of viscosity, is sufficient to prolong the life of the motion. The disturbance motion will eventually die out at all Reynolds numbers below a critical value such as 2,700. The effect at higher Reynolds numbers, wehere *U_R_*/*U_F_* progresses toward lower values, may be interpreted to mean that the initial strength was not as great as that capable of being maintained by the shear flow.

Several factors not accounted for in this paper, such as the initial strength of the disturbance, must affect the trends; therefore the diagrams should not be regarded as universal even for this particular pipe. The phenomena exhibited here are, however, believed to be universally applicable to pipe flow.

Among the foregoing phenomena, perhaps the one that has been least apparent in past experiments on pipe flow, and therefore not previously emphasized, is the one showing that below a critical value the life of a disturbance is influenced by the Reynolds number. This aspect of turbulence in pipes has usually been dismissed by stating that below the critical Reynolds number the flow will return to the laminar state no matter how much it has been disturbed. Little has been written previously on how a turbulent-like motion, when once introduced, will exist below the critical Reynolds number. Figures 8 and 9 show that the lifetime of a disturbance is prolonged more and more as the Reynolds number increases. This effect can be of some importance in practical situations where mixing is involved, either accidental or intentional, and may modify the flow field for various distances from the source of the disturbance. Just what the nature of the source might be and how disturbances might occur in practice are subjects requiring further investigation.

**Figure 10:**
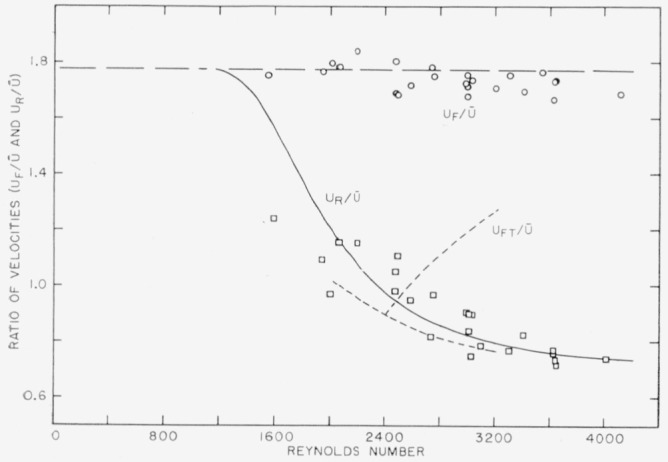
A comparison of author’s data for $U_{R}/{\bar{U}}_{F}$ with that of Lindgren (ref. 2); and for $U_{F}/\bar{U}$ with the theoretical value computed from ref. 5.

The most comprehensive previous work on the propagation and character of turbulent slugs is that of Lindgren [2, 3] conducted in straight round pipes. He used the birefringent characteristics of bentonite clay suspended in water to observe turbulent “flashes”. One aspect of his results on the velocity of the front and rear of such flashes relative to the mean-flow velocity is shown in figure 10 by the dashed curves. The results of the experiment reported here are shown by the points and the solid curve. Lindgren’s observations pertain to flashes that occurred randomly, presumably from disturbed entrance conditions, and the ones shown were made in a 6-mm tube 8.5 m from the entrance. The part of the data from this investigation, shown in the figure, pertains to the case where the dye front had traveled 15 ft from the origin. For these conditions the results for $U_{R}/\bar{U}$ obtained in the two investigations are much alike. Lindgren’s front velocity, designated here as *U_FT_*, is really the velocity of the leading end of the turbulent field. A comparable quantity, represented by the solid symbols in figures 2 and 3, could not be reliably calculated from the author’s data. The velocity of the front of the dye, *U_F_* is included in figure 10 as the ratio $U_{F}/\bar{U}$. The horizontal line is the theoretical value of $U_{c}/\bar{U}\left(=1.772\right)$ for laminar flow in the rectangular pipe as given in [5], where *U_c_* is the velocity at the center. The agreement between *U_F_* and *U_c_* shows that the dye front extended forward into the laminar regime and thus outran the turbulent field.

## 6. Conclusions

From observations of turbulent motions, created artificially at an upstream point in a rectangular pipe and allowed to pass on downstream in an otherwise fully developed laminar flow, the following conclusions are drawn:

1. It could be ascertained definitely from dye indicators that there developed a slug of turbulent pipe flow, preceded and followed by laminar flow.
2. The turbulent slug will grow indefinitely in length as it moves along the pipe if the Reynolds number of the flow is above a critical value, estimated to be 2,700. Below this critical value the growth eventually ceases.
3. Dye injected with the disturbance and also introduced as streamers is suitable for observing the rear of the slug but is poorly suited for observing the front end of the disturbance. The farthest progress of the dye, which is here called the front, is in laminar flow and moving with the centerline velocity.
4. The ratio of the velocity of the rear to the velocity of the front serves as a criterion of the vigor of the turbulent motions, and therefore is an indication of whether the motions are being sustained or are dying out as the slug moves downstream.
5. A velocity of the rear less than that of the front is taken to mean that the turbulent motions are “alive”; if the velocity of the rear increases with distance, they are dying out; if it is constant, they are maintaining their original vigor; and if it is decreasing with distance, they are growing in strength.
6. The survival time of the initially stimulated turbulent motions depends on the Reynolds number. There is a survival time below the critical value which increases as the Reynolds number increases. At the critical value and above, the survival time is infinite.

## Footnotes

2 Figures in brackets indicate the literature references at the end of this paper.
